# Mental health-related conversations on social media and crisis episodes: a time-series regression analysis

**DOI:** 10.1038/s41598-020-57835-9

**Published:** 2020-02-06

**Authors:** Anna Kolliakou, Ioannis Bakolis, David Chandran, Leon Derczynski, Nomi Werbeloff, David P. J. Osborn, Kalina Bontcheva, Robert Stewart

**Affiliations:** 1 Department of Psychological Medicine, Institute of Psychiatry, Psychology and Neuroscience, King’s College London, London, United Kingdom; 2 Department of Biostatistics and Health Informatics, Institute of Psychiatry, Psychology and Neuroscience, King’s College London, London, United Kingdom; 3 Centre for Implementation Science, Health Services and Population Research Department, Institute of Psychiatry, Psychology and Neuroscience, King’s College London, London, United Kingdom; 4 Department of Computer Science, IT University of Copenhagen, Copenhagen, Denmark; 5 Division of Psychiatry, University College London, London, United Kingdom; 6 Camden and Islington NHS Foundation Trust, London, United Kingdom; 7 Department of Computer Science, University of Sheffield, Sheffield, United Kingdom; 8 South London and Maudsley NHS Foundation Trust, London, United Kingdom

**Keywords:** Human behaviour, Epidemiology

## Abstract

We aimed to investigate whether daily fluctuations in mental health-relevant Twitter posts are associated with daily fluctuations in mental health crisis episodes. We conducted a primary and replicated time-series analysis of retrospectively collected data from Twitter and two London mental healthcare providers. Daily numbers of ‘crisis episodes’ were defined as incident inpatient, home treatment team and crisis house referrals between 2010 and 2014. Higher volumes of depression and schizophrenia tweets were associated with higher numbers of same-day crisis episodes for both sites. After adjusting for temporal trends, seven-day lagged analyses showed significant positive associations on day 1, changing to negative associations by day 4 and reverting to positive associations by day 7. There was a 15% increase in crisis episodes on days with above-median schizophrenia-related Twitter posts. A temporal association was thus found between Twitter-wide mental health-related social media content and crisis episodes in mental healthcare replicated across two services. Seven-day associations are consistent with both precipitating and longer-term risk associations. Sizes of effects were large enough to have potential local and national relevance and further research is needed to evaluate how services might better anticipate times of higher risk and identify the most vulnerable groups.

## Introduction

It has long been recognised that material circulated via news media can have important mental health-relevant outcomes. Research in this area has particularly focused on suicide or non-fatal self-harm carried out by well-known people or fictional portrayals of these issues in widely viewed sources, addressing concerns about imitative behaviour^1^. In parallel, there has also been concern around potentially stigmatising material and its longer-term impacts on the wellbeing of people with mental disorders and those who care for them^2^. Depictions of mental illness on television and in film^3^ and newspaper coverage of high-profile suicides^4^ can have profound implications not only for public health and opinion but also directly on those experiencing the mental health issues portrayed^5^. The explosion of social media channels and record levels of public use^6^, round-the-clock access to breaking news, stories and discussions as well as the sheer volume of decreasingly controlled information have transformed and amplified exposure to mental health dialogues and representations of mental illness.

The use of data-rich, novel sources such as results from internet searches, social media communications and environmental records is becoming a potentially fast and cost-effective way to identify population needs and predict or prevent healthcare emergencies, such as pharmacovigilance^7^. More recently, information from Twitter has been utilised for analysing behaviours, attitudes and experiences related to mental health^8^, particularly depression^9,10^. In parallel, the expansion of electronic health records (EHRs) has offered extensive, longitudinal clinical information from large groups of service users, which have routinely been used to facilitate patient care^11^ and support public health monitoring and intervention^12^.

Temporal relationships between media events and mental health outcomes have to date primarily focused on high-profile events extracted from news media but associations with the fluctuating prominence of mental health-relevant issues on social media have remained relatively under-investigated. Taking advantage of novel ‘big data’ from both mental healthcare and social media and funded by PHEME, a European Commission consortium working to identify misinformation in social media (www.pheme.eu) particularly that relating to mental health, we sought to investigate the extent to which day-to-day fluctuations in Twitter discussions about two important mental health disorders (depression and schizophrenia) would be associated with mental health crisis episodes in secondary mental healthcare. On the assumption that Twitter content reflects wider awareness and conversation beyond simple news stories (and might be a better marker of the penetrance of news stories into public consciousness and discussion and thus the exposure as experienced by those with mental disorders), we set out to test a hypothesis that a higher number of mental health-related tweets would be associated with higher numbers of crisis episodes on the same day over a four-year period. We also conducted an exploratory time-lagged analysis to investigate the association between tweets and crisis episodes on days following shortly after. This time-lagged analysis was not hypothesis-driven; however, we did seek to clarify the consistency of an observation in one EHR data resource by testing it in an independent site and sample.

## Results

Over the 5-year period, 48,691 crisis episodes were recorded in South London and Maudsley NHS Foundation Trust (SLAM) (median 28 per day; IQR: 3 to 68) and 32,689 episodes in Camden and Islington NHS Foundation Trust (C&I) (median 19, IQR: 1 to 57). Median (IQR) tweet numbers per day were: 6 per 10 million (0 to 50) for general depression; 10 per 1 billion (0 to 152) for general schizophrenia; 0 per 10 billion (0 to 295) for supportive depression; and 0 per 10 billion (0 to 29) for supportive schizophrenia. Total numbers of above-median days were: 920 for general depression, 901 for general schizophrenia, 974 for supportive depression and 461 for supportive schizophrenia and Supplementary Figs. 1–4 provide a graphical representation of tweet distribution over time. In order to ascertain potential clustering of above-median days, distributions by week are displayed in Supplementary Table 1. While general depression and general schizophrenia tweets showed a preponderance for clustering (shown by the high proportions of weeks containing seven above-median days), the supportive depression/schizophrenia tweets showed a more even distribution. Median (IQR) daily temperature was 10 °C (−7 to 22), and high (over 100%) bed occupancy level was recorded on 16% of days (n = 291) for SLAM and on 100% of days (n = 1,826) for C&I. The occupancy level covariate was therefore dropped from the C&I adjusted regression models. Further descriptive data are presented on exposures and SLAM outcome by day of the week in Supplementary Table 2. The median number of crisis episodes in SLAM showed an increase from Monday to Friday with lower levels over the weekend; however, mental health tweet measures were more evenly distributed throughout the week without distinct peaks.

**Figure 1:**
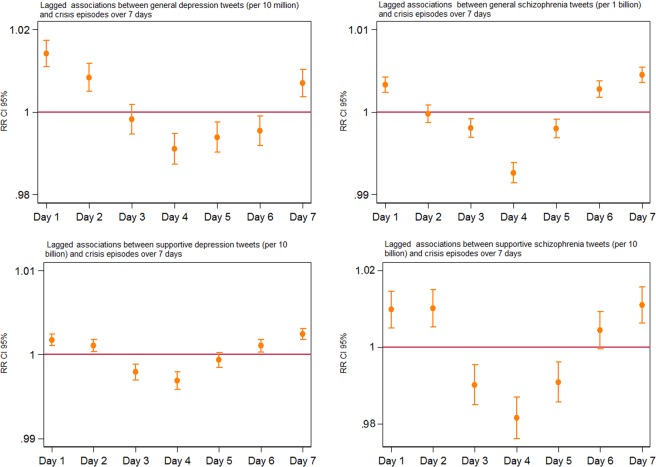
Lagged associations between mental health tweets and SLAM crisis episodes adjusted for autocorrelation, year, temperature, seasonality and occupancy level. The horizontal axis represents the associations lagged over a 7-day period and the vertical axis represents the Relative Risks (RR) with 95% confidence intervals (CI).

Analyses of contemporaneous associations between tweet volumes and same-day crisis episodes showed positive associations in SLAM for all four measures of mental health-related tweet content and near-identical coefficients when the same models were run using C&I data (Table 1). The step-wide adjustment process for both sites can be found in Supplementary Table 3. The use of the 7-day lag model showed a curvilinear pattern of associations between mental health-related tweets and crisis episodes in SLAM after adjustment for autocorrelation, year, temperature, seasonality and occupancy level (Fig. 1). Patterns of association were similar for all four measures of tweet content, with initial positive associations on Day 1, moving to negative associations by Day 4 and followed by a return to positive associations by Day 7. Near-identical patterns were observed when the same lag model was applied to C&I data (Supplementary Fig. 5).

**Table 1:** Concurrent unadjusted and adjusted associations between daily tweet volumes and daily crisis episodes at the participating sites.

Tweet content	Crisis episodes SLAM		Crisis episodes C&I	
	Unadjusted^ RR (95% CI)	Adjusted^†^ RR (95% CI)	Unadjusted^ RR (95% CI)	Adjusted^¥^ RR (95% CI)
Depression - general^a^	1.003 (1.000–1.007)	1.008* (1.002–1.014)	1.000 (0.934–1.003)	1.008* (1.001–1.015)
Schizophrenia - general^b^	1.003** (1.002–1.004)	1.006** (1.004–1.008)	1.002* (1.000–1.003)	1.006** (1.003–1.008)
Depression - supportive^c^	1.002** (1.001–1.003)	1.003** (1.001–1.004)	1.003** (1.001–1.004)	1.003** (1.001–1.005)
Schizophrenia - supportive^d^	1.014** (1.006–1.021)	1.015** (1.010–1.022)	1.012** (1.004–1.020)	1.014** (1.006–1.022)

Relative risks (RR) and 95% confidence intervals (CI) represent an increased risk of crisis episodes per unit increase in tweet volume.

^**^**^Adjusted for autocorrelation only.

^**†**^Adjusted for autocorrelation,year, temperature, seasonality and occupancy level.

^¥^Adjusted for autocorrelation, year, temperature and seasonality.

*p < 0.001.

**p < 0.005.

^a^per 10 million.

^b^per 1 billion.

^c^per 10 billion.

^d^per 10 billion.

Based on a median split, days when more than 6 per 10 million and 10 per 1 billion tweets were posted referring to general depression and schizophrenia, respectively, were considered higher volume days, as were days when any supportive depression and schizophrenia tweets were published. After full adjustment for autocorrelation, year, temperature, seasonality and occupancy level, SLAM crisis episodes were 15% higher (p < 0.001) on higher-volume schizophrenia tweet days, 9% higher (p < 0.001) on higher-volume supportive depression tweet days and 10% higher (p < 0.001) on higher-volume supportive schizophrenia tweet days (Supplementary Table 4). To ensure that the delayed associations shown in the 7-day lag model were not affected by fluctuations due to specific days of the week, two sensitivity analyses repeated the lag models for 5 and 3 days; in summary, sizes and directions of associations remained largely unchanged (Supplementary Figs. 6 and 7).

In a final exploratory analysis investigating the likely relationship between Twitter and wider media coverage on the topics of interest, we investigated the level of overlap between our Twitter data and those derived from Google Trends. By identifying the highest two peaks in each of our mental health-related tweet groups and using the news search function on Google Trends (worldwide, date range 01/01/2010 to 01/01/2015, health category, news search, keywords: depression, schizophrenia, stigma), we were able to confirm peaks in news searches during the preceding or concurrent week that corresponded to the peaks in our Twitter dataset (Supplementary Table 5).

## Discussion

Bringing together large mental healthcare and social media data resources, we investigated the extent to which mental health-related posts on Twitter were associated with contemporaneous and subsequent crisis episodes reported in mental health services. Our findings supported hypothesised associations and we were able to replicate them near-identically in an independent clinical sample.

A relationship has long been suspected between public events relating to mental health and personal crises in vulnerable populations hearing about these. For example, there have been a number of studies of temporal links between the suicide-related deaths of public figures and suicidal behaviour recorded in administrative data from exposed populations^4,5^. It is therefore assumed that at least some people are sensitised to public events and many nations have specific guidance and/or protocols to which their national media are expected to adhere^13,14^ which has resulted in at least some improvement in coverage over time, albeit mixed in some circumstances^15^. In addition, a large body of research has investigated perceived stigma in people with mental health disorders, its prognostic associations and interventions to counter its influence^16,17^. Furthermore, increasing attention is being paid to the ways in which individuals’ mental health status can be inferred from material on social media^18^ and predicted from previous material^19^. Our exploratory findings of relationships between peak Twitter and Google Trends for news searches supports the notion that Twitter reflects mental health-related events in the wider news-sphere. However, despite the growth in these fields of enquiry, there has been little investigation, to our knowledge, of temporal relationships between phenomena measured on social media platforms and administrative data relevant to mental health. This is what we sought to estimate in our study.

Our hypothesis and the predictions being tested in these analyses relied on measuring daily levels of relevant Twitter posts and daily levels of crisis episodes. We anticipated that there would be considerable measurement error in both of these constructs, since Twitter posts are likely to be only a proxy indicator of exposures potentially influencing mental health, and mental healthcare crisis episodes are only a proxy indicator of population mental health. Furthermore, we were not able to characterise social media exposures into those most likely to have a mental health impact and we were not able to focus crisis episode measurements on to groups most vulnerable to these exposures. Our findings should therefore be viewed as a likely underestimate of the association of interest and the observed strength is therefore important. For example, the observed 5–15% increases in crisis episodes on days with higher (above-median) relevant Tweet proportions represent quite substantial volumes and costs of service activity, if they were to be present across all mental health services nationally. This effect size is in line with a recent study which found an almost 10% increase in general population suicides (16,849 expected, 18,690 actual) during the 6 months following the death of Robin Williams^20^.

The pattern of lagged associations was originally derived from an exploratory analysis in the SLAM dataset, and was not one that was originally hypothesised; however, it was re-evaluated in the independent data provided by C&I and was found to be surprisingly consistent across both clinical datasets. Underlying reasons for the pattern require further investigation; however, a possible explanation for the initial positive association on day 1 followed by a negative association by day 4 is that crisis episodes which might have happened anyway over that period were precipitated early (i.e. someone who would under other circumstances have experienced their crisis on day 4 had presented instead on day 1). On the other hand, the return to a positive association by the end of the 7-day lag period would be consistent with a more prolonged higher risk period associated with the exposure. Of note, although a sizeable number of days with above-median depression/schizophrenia tweet volumes were clustered (e.g. in the weeks with seven above-median days described in Supplementary Table 1), the 7-day pattern of the association was present also for days with higher numbers of supportive tweets, which were more evenly distributed. Both precipitating and longer-term effects are potentially important as indicators of distress caused to individuals and as reflections of higher mental health service activity as a consequence.

Strengths of the study include the large sample sizes and the ability to replicate findings in an independent dataset, as has been employed in previous multi-site studies using CRIS-derived mental healthcare data^21^. Considering potential alternative explanations for observed associations, we believe that we were able to exclude the most important. By measuring mental health-related tweets as a proportion of total tweets, we excluded potential associations with overall rather than specific post volumes. In addition, although there were weekly variations in crisis episodes, as has been reported for other mental health outcomes in our data^22^, this was not markedly the case for relevant tweet volumes and the sensitivity analyses excluded a day-of-the-week effect. Seasonality and temperature were likewise considered and excluded as confounders, as was variation in bed occupancy levels.

Limitations primarily concern the ability to infer causal pathways. Direct reverse causality (crisis episodes generating higher Twitter activity) is unlikely, because the impact of two UK services on Twitter post volumes would be negligible; however, it is not assumed that the content of the tweets themselves was directly influencing the likelihood of crisis, as we do not know the level of exposure to Twitter in those experiencing crises. Rather it is more likely that it is the source events generating the higher tweet volumes which are influencing mental health, potentially amplified by their dissemination through social media platforms, although this would require further research. Similarly, the observed risk associations with ‘supportive’ tweets are likely to be accounted for by the negative material which these tweets are intending to counter rather than the supportive tweets themselves. Although it seems likely that the dissemination of specifically stigmatising material plays some role in our observed associations with crisis episodes, this would also need to be investigated empirically. In addition, while we chose depression and schizophrenia as indicator mental health conditions for screening Twitter posts, we did not seek to investigate the specificity of associations (i.e. whether a day with higher schizophrenia-related tweets was associated primarily with crisis episodes in people with that particular diagnosis). Furthermore, we used the same exposure (tweet volumes) for the different SLAM and C&I databases. A future study with the use of geo-located tweets stemming from the areas under study may provide a better insight; however, although this method could track what tweet volumes are being posted from a specific area, it would not provide any clearer picture of the reading patterns of the population under study.

Social media use is increasingly being cited as associated with poor mental health in young and older adults alike. Patterns of ‘problematic’ use have been shown to increase depression and anxiety symptoms and affect a user’s psychological state through emotional contagion (emotional states being involuntarily transmitted between individuals)^23,24^. Negative impact on users’ psychological wellbeing, from anxiety to self-esteem, has also been evidenced among adolescents^25^. Finally, although social networking sites are widely used to promote interpersonal relationships, research suggests that their use by individuals with narcissistic traits provides opportunities for self-promotion and anti-social behaviour that negatively affects the wellbeing of others^26^. We do believe that our own findings contribute some support to concerns, already widely expressed^27^, about mental health conversations on Twitter. While it is possible that source news stories were the primary stressors here and we were not able to investigate formally any amplifying or distorting effect of discussions on social media which would require more specific empirical investigation for mental health issues, it is undoubtedly the case that it is less easy to intervene in these less regulated environments than it is to promote good journalistic practice in the portrayal of mental health issues in mainstream media. Further research, most likely requiring mixed qualitative and quantitative approaches is also required to evaluate this further, particularly identifying populations who are most vulnerable and developing and evaluating interventions whether delivered at an individual level in mental healthcare or at a national/international level through trying to counter the messages most likely to be negative. In the meantime, given the relatively sizeable effects visible at a service level, there may be a good reason to consider setting up a monitoring system to identify risk days and communicate these to mental health and other relevant services, at least to forewarn staff to potential effects and promote support for service users.

## Methods

### Setting

Data from two mental health services were used in this study. SLAM is one of the largest mental healthcare providers in Europe, delivering comprehensive services to a catchment population of around 1.3 million residents in four south London boroughs (Lambeth, Southwark, Croydon and Lewisham). C&I is a large mental healthcare provider offering services to approximately 470,000 residents in a geographic catchment area of two north London boroughs (Camden and Islington). All analyses were carried out to completion using SLAM data first. Replicability of findings, particularly those derived from exploratory analyses on SLAM data, was then assessed using C&I data as an independent source.

### Data sources and extraction

Clinical data for this study were obtained first from the SLAM Biomedical Research Centre (BRC) Case Register: a repository of anonymised clinical data from the electronic health records (EHRs) of individuals receiving care from SLAM mental health services^28^. The register contains, at the time of writing, over 350,000 de-identified patient records which are available for research purposes through the Clinical Record Interactive Search (CRIS) application^29^. CRIS was developed at SLAM in 2008 and has subsequently been implemented at several other mental health Trusts, including C&I whose research database contains de-identified information on more than 108,000 patients^30^. The CRIS tool has been approved for secondary analysis (for SLAM: Oxford C Research Ethics Committee, reference 08/H0606/71 + 5; for C&I: NRES Committee East of England - Cambridge Central, reference 14/EE/0177) and all proposed research requires approval by a patient-led oversight committee at each site before access to data is authorised^30,31^.

Twitter, one of the largest social media sources, is a micro-blogging platform which, as of the fourth quarter of 2018, has around 321 million active users^32^. On average, around 6,000 tweets are posted on Twitter every second, which corresponds to around 200 billion tweets per year making this source exceptionally rich in text, graph, and video and image interaction^33^.

For this study, we had access to a random 10% sample of all tweets published from January 2010 to December 2014 inclusive, archived from a Twitter feed collected by the University of Sheffield. We applied an iterative process to organise the list of keywords (Supplementary Table 6) used to search the tweet sample. With our focus on depression, schizophrenia and mental health stigma, we tested a number of terms (depression, depressed, depressing, schizophrenia, schizoaffective, schizophrenic, schizo, stigma, mental illness, mental health) related to these disorders by using the Twitter search function (twitter.com/search-home?lang = en-gb). ‘Schizophrenia’ here included the broader diagnostic construct of schizophreniform disorders such as schizoaffective disorder. We then checked how they appeared in about 100 tweets per term (i.e. Does it truly relate to the subject? Is it used flippantly? Does it refer to human emotional states or other animals/inanimate objects? Is it commonly used as a hashtag?) and subsequently held consensus meetings to discuss these findings and finalise the keyword list. There was noted to be a high number of references to mental health stigma using hashtags (#stigma, #stigmahurts etc), a pattern that was only observed, in the two disorders, for the derogatory term ‘schizo’. Tweets that contained stigma-related hashtags usually included the ‘mentalhealth’ or ‘mentalillness’ hashtag too. It thus appeared that users were unlikely to use hashtags for mental health issues they were sharing from personal experience; however, the use of hashtags was very common when referencing derogatory terms or discussing more socially expansive issues such as stigma. The final list was used to query the tweet sample by using a pre-defined search^34^ first to reduce the number of records processed in detail. The remaining records were then de-serialised, non-English tweets were filtered out by language identification where available, and the terms were searched for in just the tweet text field. All the above processes were performed on a Sun Grid Engine cluster. Through this process, 7,121,043 and 134,653 tweets relating to depression and schizophrenia, respectively, were retrieved. The extracted tweets were read, as in one per line, in Microsoft Excel. These extractions are part of a wider Twitter content analysis under the PHEME project examining mental health disorders, mental health stigma, self-harm and suicide and psychotropic medications. The separate codes for each search and extraction can be found at github.com/leondz/medtermfilter. Another investigation under PHEME using a similar methodology and examining novel psychoactive substances in the EHR and online data sources has also been described elsewhere^35^.

As we were preparing to compile the three datasets related to the above searches and subsequently work on developing an algorithm for the identification of tweets related to perceived stigma, the Germanwings Flight 9525 crash occurred. The event generated a degree of controversy and conversation concerning the mental health of the co-pilot, who was suspected of causing the crash as a suicide act. Therefore, we decided to focus the development of the stigma algorithm on the analysis of Twitter content between March 23 and March 30 2015, covering the date of the event and the following 7 days instead of using the dataset already collected. A new query using the search terms *depress*, mental* and psych* during this period produced 20,713 tweets. Using 2,000 tweets, good inter-annotator agreement between two coders was obtained (kappa 0.79; 95% CI 0.76, 0.81) and a random split-half approach (1,000 training tweets; 1,000 test tweets) was used to generate an algorithm with a precision score (positive predictive value) of 0.98 and a recall score (sensitivity) of 0.31 using the Generalised Architecture for Text Engineering (GATE)^36^ platform. The algorithm was designed to detect tweets which could be classified as ‘supportive’, i.e. those seeking to counter perceived stigma, since these had been found to be more tractable for stigma detection than attempting to search for potentially stigmatising material itself. Applying this algorithm over the larger set of tweets (7,255,696) retrieved between 2010 and 2014, 5,003 and 847 supportive tweets were classified relating to depression and schizophrenia, respectively.

### Outcomes

For the analysis, the daily number of incident admissions to inpatient and Home Treatment Team (HTT) services (and for C&I, Crisis House admissions) was extracted using CRIS for both Trusts between January 1 2010 and December 31 2014. An incident admission was defined as a recorded new admission to either one of these services commencing on each day in question. Patients admitted to and discharged from services on the same day were included in the sample.

### Temporal confounders

The Hadley Central England Temperature (HadCET)^37^ dataset was used to extract daily temperatures. The mean daily temperatures, available since 1772, are representative of a roughly triangular area of the UK enclosed by Lancashire, London and Bristol. We controlled for temperature as a proxy for seasonality which has been considered to affect psychiatric admissions^38^.

The daily numbers of occupied overnight beds in SLAM and C&I were obtained from structured data extracted via CRIS for both Trusts. The Trusts do not operate beds for daytime-use only and do not hold an official record for available overnight beds. Hence, the daily numbers of available overnight beds for each Trust were obtained from NHS England which collects this information quarterly^39^. From these data, we produced a daily occupancy rate and dichotomised it to high (≥100%) and low (<100%) levels: again, as an assumed time-varying exposure which would also influence daily admission rates.

We also controlled for calendar year as availability of beds has been steadily declining in the UK during the last 2 decades^40^.

### Statistical analysis

All statistical analyses were performed using STATA 13.1. Descriptive statistics were obtained as medians and inter-quartile ranges (IQRs) for crisis episodes, tweets and temperature, and as percentages for occupancy level. Analyses were also carried out to describe the degree of clustering of days with high-volume tweets (by describing the distributions of days-per-week implicated in this respect), the variation by day-of-the-week in both tweet and crisis episode measures, and the distribution of tweets of interest (graphically by time). Contemporaneous and concurrent associations between mental health-related tweets and crisis episodes on each day were assessed with Poisson regression models. All Poisson regression models were adjusted for temperature, daily number of occupied beds and calendar year and a long-term pattern of seasonality fitting a Fourier term to account for monthly seasonal trends. We also took into account autocorrelation between our daily observations with the use of Newey-West standard errors^41^. We envisaged that the association might be immediate (contemporaneous) or might occur with some delay (lagged); therefore we created time-shifted copies of our exposure variables and included them in the Poisson regression models to examine delayed mental health-related tweet effects (one lag = one day) on mental health crisis episodes over a 7-day period. Because a significant lagged association could be due to a particular day of the week when crisis episodes may be consistently higher, we conducted two sensitivity analyses by repeating the Poisson regression analysis for a 3-day lag and a 5-day lag. Finally, in order to quantify the potential service impact of associations observed on mental health crisis episodes, we dichotomised each of the tweet measures into above- and below-median daily levels and re-calculated associations with crisis episodes.

## Supplementary information

Supplementary tables and figures.

## Data Availability

The datasets during and/or analysed during the current study are available from the corresponding author on reasonable request.
