# Supplementary material for: Mental health-related conversations on social media and crisis episodes: a time-series regression analysis
Source: Sci Rep. 2020 Feb 6;10:1342. doi: 10.1038/s41598-020-57835-9 (PMC7005283; doi:10.1038/s41598-020-57835-9)
Supplement: Supplementary file 1 — Supplementary tables and figures. [file 41598_2020_57835_MOESM1_ESM.pdf]

Mental health-related conversations on social media and crisis episodes: a time-series regression analysis.

Anna Kolliakou, Ioannis Bakolis, David Chandran, Leon Derczynski, Nomi Werbeloff, David PJ Osborn, Kalina Bontcheva, Robert Stewart

Tweet group	Number (%) of weeks with a given number of days containing above-median numbers of tweets							
	**0 days**	**1 day**	**2 days**	**3 days**	**4 days**	**5 days**	**6 days**	**7 days**
General depression	99 (38.1)	14 (5.4)	6 (2.3)	10 (3.8)	10 (3.8)	8 (3.1)	10 (3.8)	103 (39.6)
General schizophrenia	70 (26.9)	32 (12.3)	17 (6.5)	14 (5.4)	14 (5.4)	17 (6.5)	21 (8.1)	75 (28.8)
Supportive depression	19 (7.3)	28 (10.8)	38 (14.6)	34 (13.1)	42 (16.2)	33 (12.7)	27 (10.4)	39 (15.0)
Supportive schizophrenia	76 (29.2)	71 (27.3)	43 (16.5)	23 (8.8)	13 (5.0)	25 (9.6)	5 (1.9)	4 (1.5)

Supplementary Table 1. Distribution of days with above-median mental health-related tweets for 260 analysed weeks between 01/01/2010 and 31/12/2014.

Day of the week	median (IQR)				
	**SLAM crisis episodes**	**General depression tweets (per 10 million)**	**General schizophrenia tweets (per 1 billion)**	**Supportive depression tweets (per 10 billion)**	**Supportive schizophrenia tweets (per 10 billion)**
Monday	28 (5-65)	6 (0-23)	9 (0-54)	1 (0-112)	0 (0-11)
Tuesday	32 (7-50)	7 (0-18)	11 (0-151)	1 (0-129)	0 (0-14)
Wednesday	31 (7-50)	5 (0-49)	11 (0-114)	2 (0-295)	0 (0-19)
Thursday	32 (7-68)	6 (0-23)	10 (0-70)	2 (0-65)	0 (0-29)
Friday	35 (11-56)	5 (0-21)	10 (0-53)	0 (0-38)	0 (0-10)
Saturday	16 (4-35)	6 (0-16)	9 (0-67)	0 (0-27)	0 (0-11)
Sunday	11 (3-21)	6 (0-39)	8 (0-69)	0 (0-57)	0 (0-10)

Supplementary Table 2. Descriptive data for SLAM outcome and exposures by day of the week between Jan 1st 2010 and Dec 31st 2014.

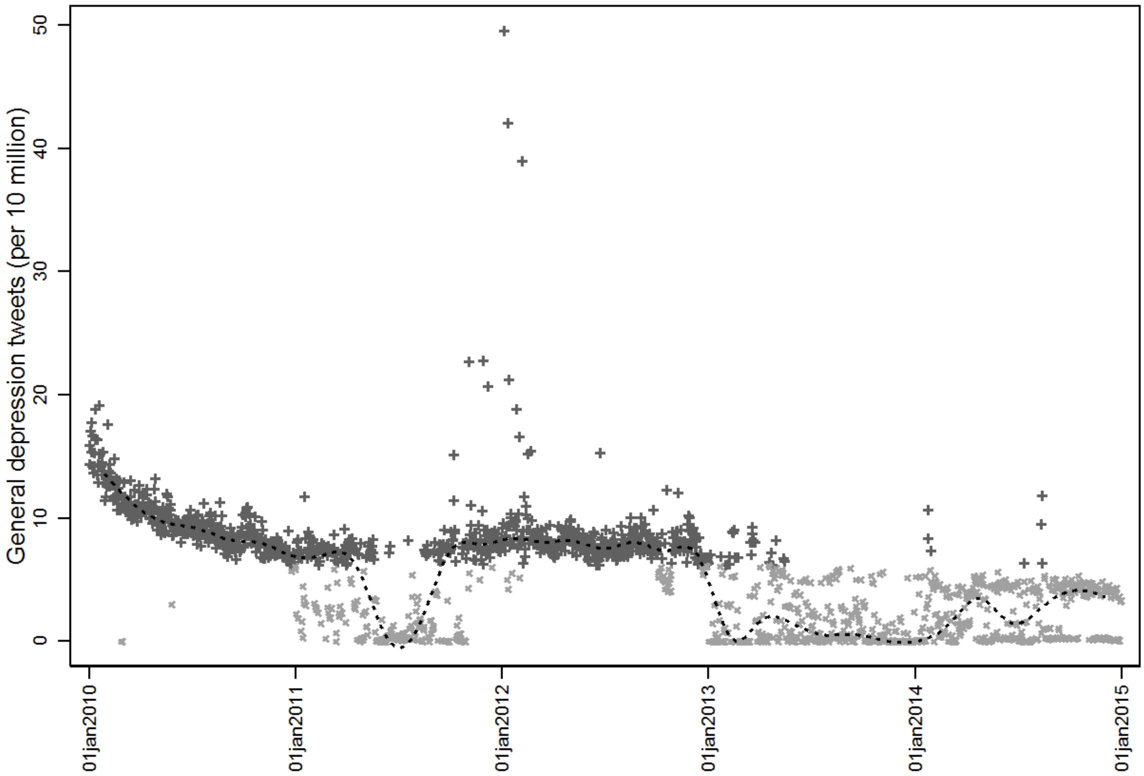

Supplementary Figure 1. Distribution of general depression tweets between 2010 and 2015.
+ above-median tweet day
- below-median tweet day
- - - trend line

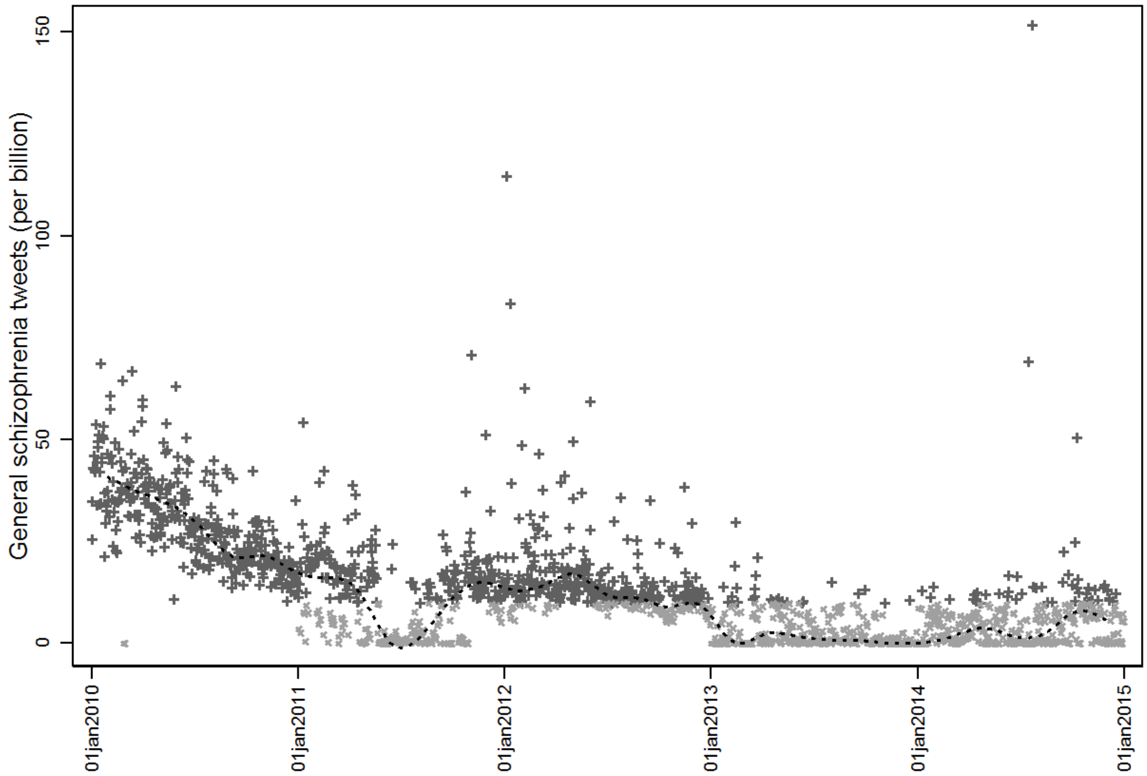

Supplementary Figure 2. Distribution of general schizophrenia tweets between 2010 and 2015.
+ above-median tweet day
- below-median tweet day
- - - trend line

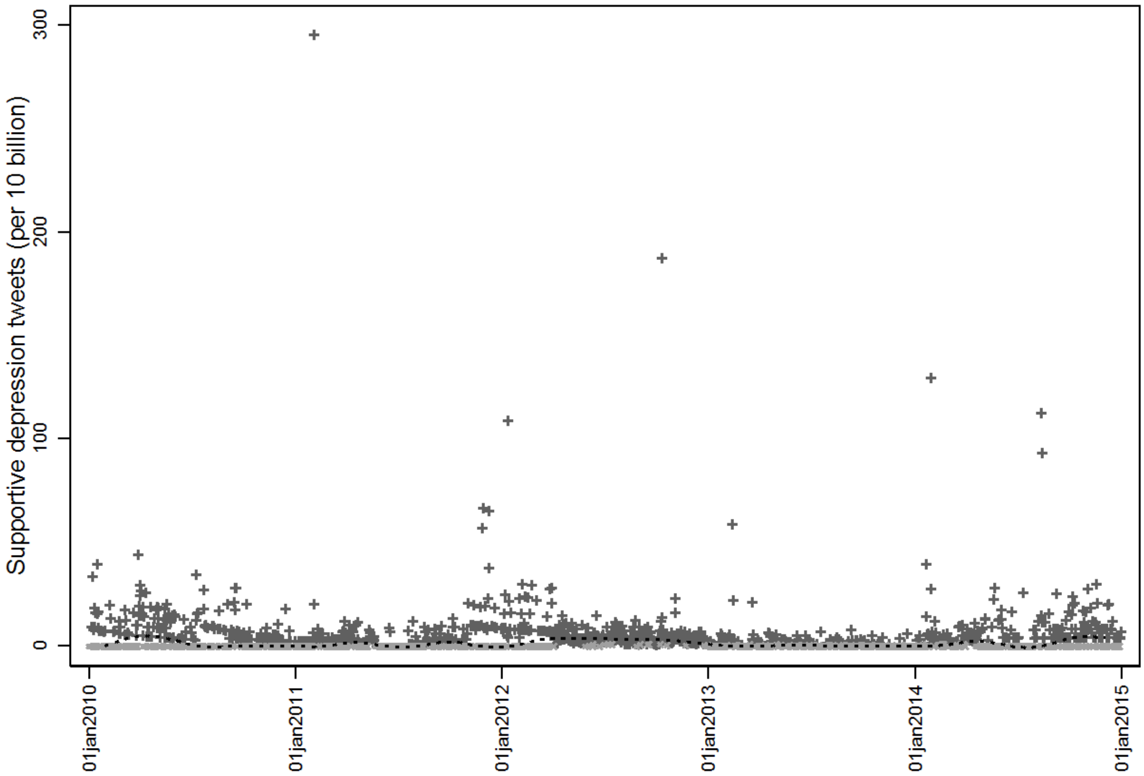

Supplementary Figure 3. Distribution of supportive depression tweets between 2010 and 2015.
+ above-median tweet day
- below-median tweet day
- - - trend line

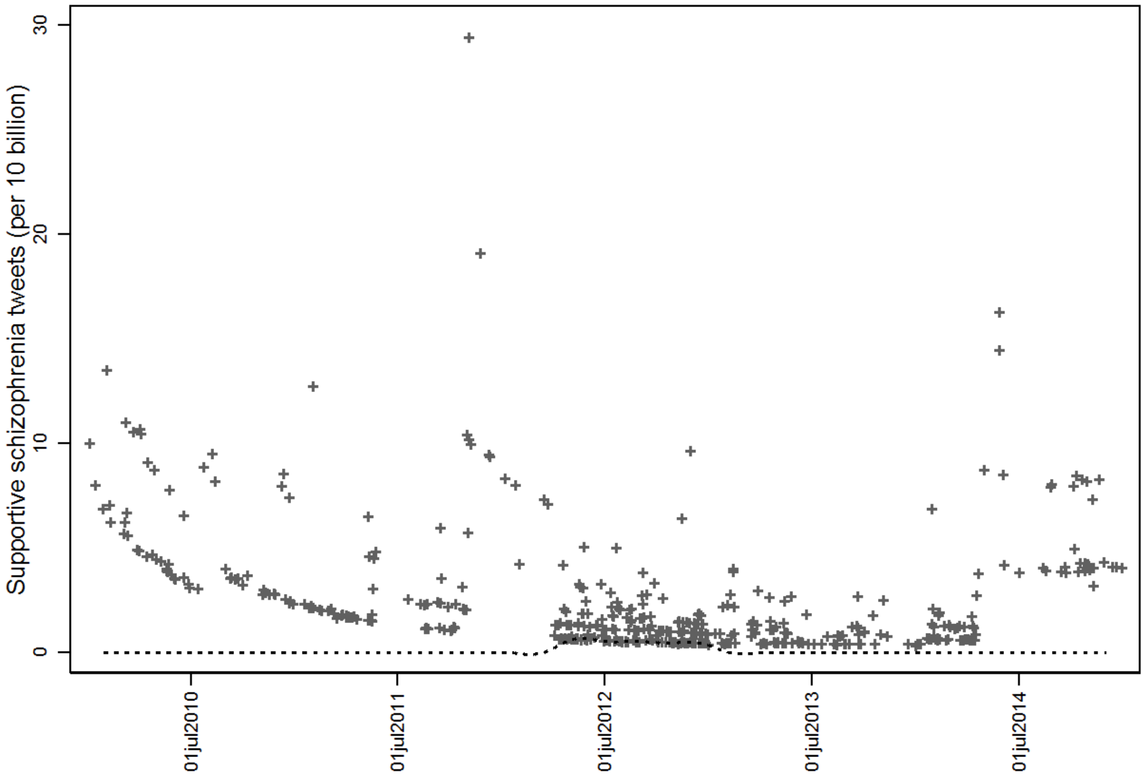

Supplementary Figure 4. Distribution of supportive schizophrenia tweets between 2010 and 2015.
+ above-median tweet day
- below-median tweet day
- - - trend line

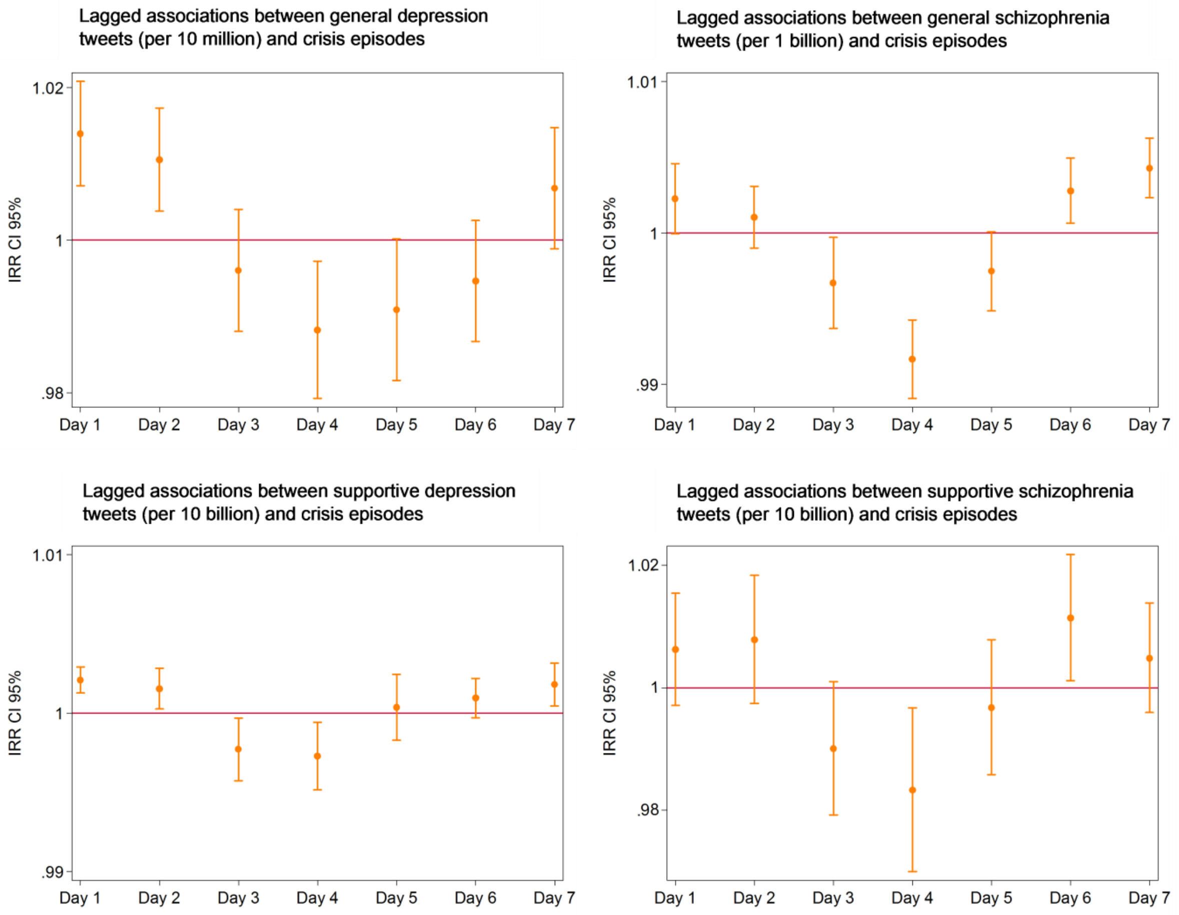

Supplementary Figure 5. Lagged associations between mental health tweets and C&I crisis episodes adjusted for autocorrelation, year, temperature and seasonality. The horizontal axis represents the associations lagged over a 7-day period and the vertical axis represents the Relative Risks (RR) with 95% confidence intervals (CI).

**Tweet group**	**Unadjusted**	**Adjusted**<br>Year	**Adjusted**<br>Year<br>Temperature	**Adjusted**<br>Year<br>Temperature<br>Occupancy^	**Adjusted**<br>Year<br>Temperature<br>Occupancy^<br>Seasonality
**SLaM**	**RR(95% CI)**	**RR(95% CI)**	**RR(95% CI)**	**RR(95% CI)**	**RR(95% CI)**
General depression[1]	1.003^{-} (0.999-1.007)	1.006** (1.000-1.012)	1.007** (1.001-1.013)	1.007** (1.001-1.013)	1.008** (1.002-1.014)
General schizophrenia[2]	1.003** (1.002-1.004)	1.005** (1.003-1.008)	1.006** (1.004-1.008)	1.006** (1.003-1.008)	1.006** (1.004-1.008)
Supportive depression[3]	1.002** (1.001–1.003)	1.002** (1.001-1.003)	1.002** (1.001-1.003)	1.002** (1.001-1.004)	1.003** (1.001-1.004)
Supportive schizophrenia[4]	1.014** (1.006-1.021)	1.014** (1.006-1.022)	1.014** (1.007-1.022)	1.014** (1.007-1.022)	1.015** (1.007-1.023)
**C&I**	**RR(95% CI)**	**RR(95% CI)**	**RR(95% CI)**	**RR(95% CI)**	**RR(95% CI)**
General depression[1]	1.000^{-} (0.993-1.003)	1.006^{-} (0.999-1.013)	1.008* (1.001-1.015)	As previous step	1.008** (1.001-1.015)
General schizophrenia[2]	1.003** (1.002-1.004)	1.006** (1.003-1.008)	1.006** (1.003-1.008)	As previous step	1.006** (1.004-1.008)
Supportive depression[3]	1.002** (1.001-1.003)	1.002** (1.001-1.004)	1.002** (1.001-1.004)	As previous step	1.003** (1.001-1.004)
Supportive schizophrenia[4]	1.014** (1.006-1.021)	1.014** (1.007-1.022)	1.014** (1.007-1.022)	As previous step	1.015** (1.007-1.023)

Relative risk (RR) and 95% confidence intervals (CI) represent an increased risk of crisis episodes per unit increase in tweet volume
- not significant
* p<0.001
** p<0.005
^ SLAM-only
[1] per 10 million
[2] per 1 billion
[3] per 10 billion
[4] per 10 billion

Supplementary Table 3. Confounder adjustment process in the concurrent unadjusted and adjusted associations between daily tweet volumes and daily crisis episodes in SLAM and C&I.

Tweet content	SLAM Crisis Episodes<br>RR (95% CI)	P value
General depression	1.04 (0.98-1.11)	0.181
General schizophrenia	1.15 (1.10-1.21)	<0.001
Supportive depression	1.09 (1.05-1.14)	<0.001
Supportive schizophrenia	1.10 (1.06-1.15)	<0.001
Adjusted for autocorrelation, year, temperature, seasonality and occupancy level		

Supplementary Table 4. Adjusted associations between above-median tweet volume days and daily crisis episodes.

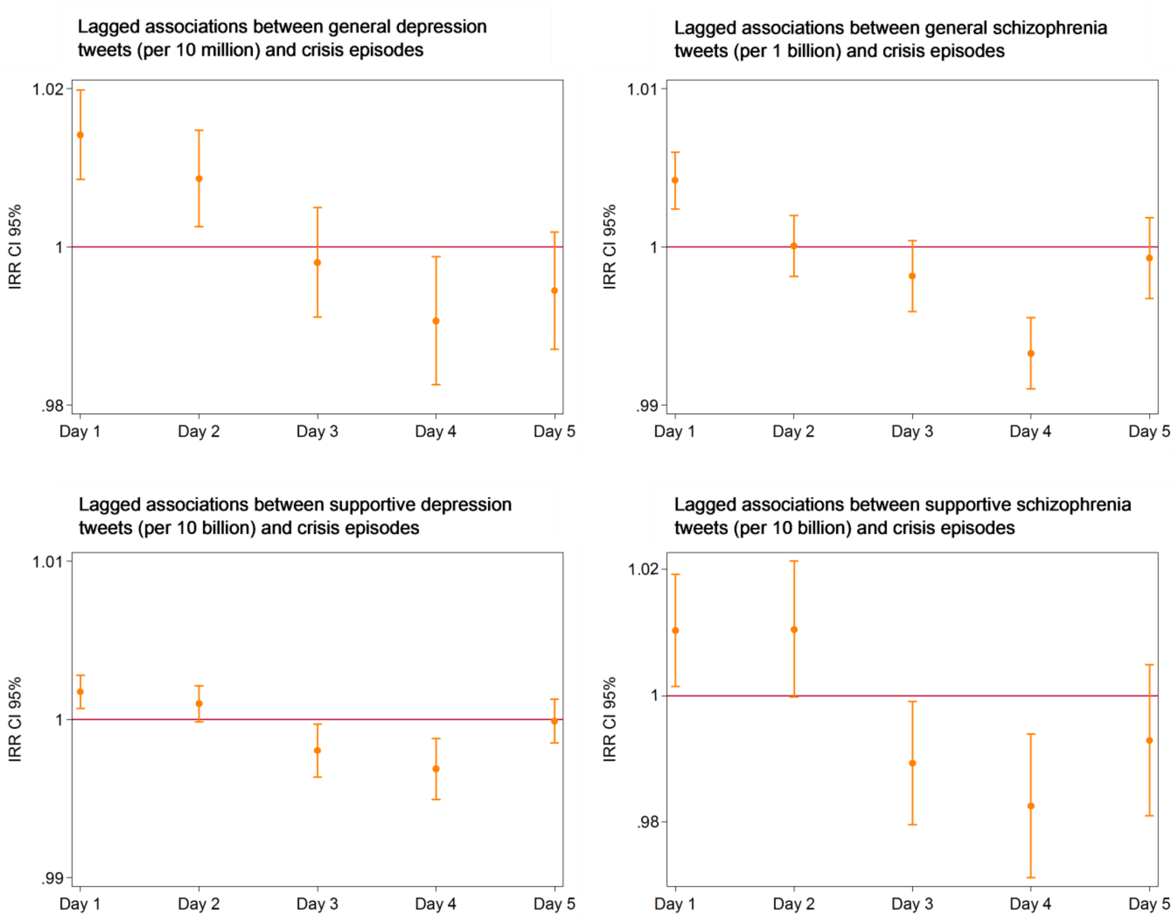

Supplementary Figure 6. Lagged associations between mental health tweets and SLAM crisis episodes adjusted for autocorrelation, year, temperature, seasonality and occupancy level. The horizontal axis represents the associations lagged over a 5-day period and the vertical axis represents the Relative Risks (RR) with 95% confidence intervals (CI).

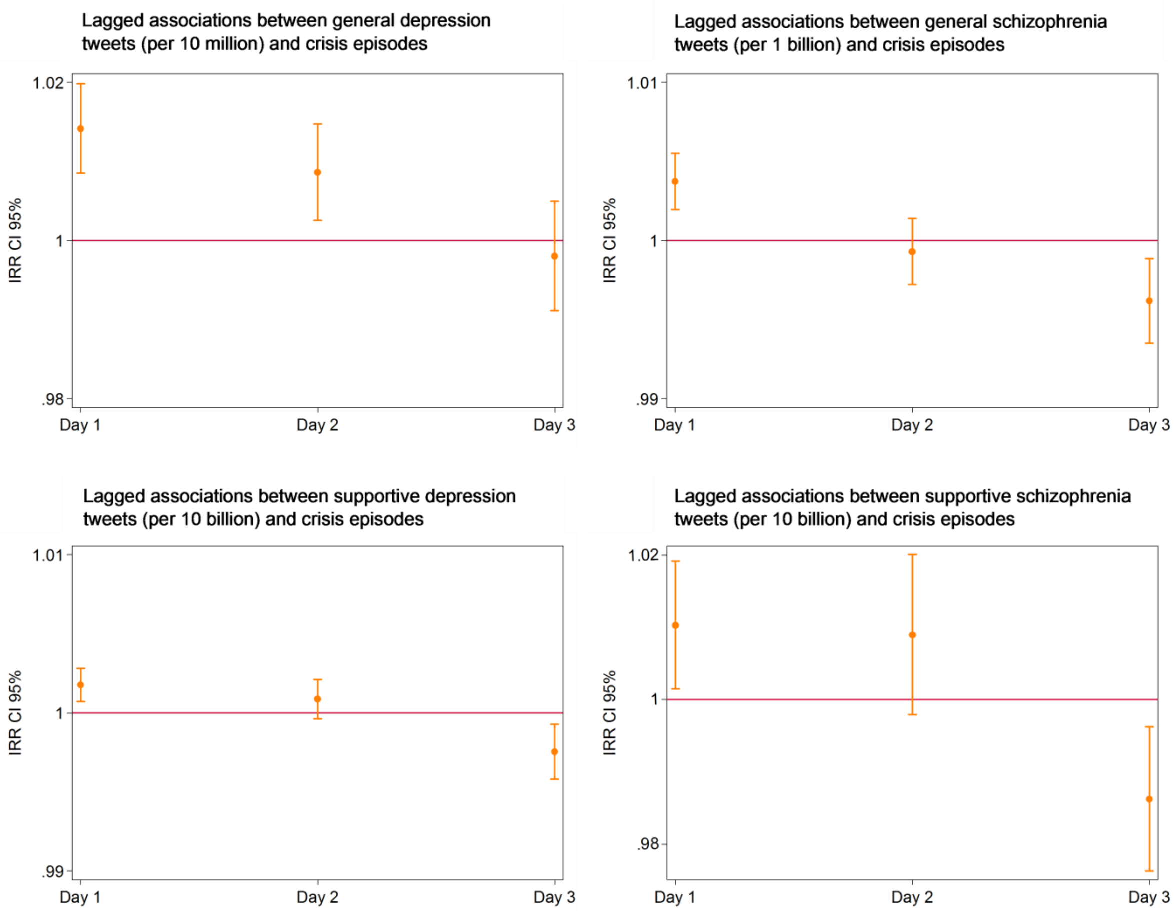

Supplementary Figure 7. Delayed associations between mental health tweets and SLAM crisis episodes adjusted for autocorrelation, year, temperature, seasonality and occupancy level. The horizontal axis represents the associations lagged over a 3-day period and the vertical axis represents the Relative Risks (RR) with 95% confidence intervals (CI).

Tweet group	Twitter peak	Google Trends peak
General depression	04/01/2012<br>11/01/2012	01-07/01/2012
General schizophrenia	04/01/2012<br>22/07/2014	25-31/12/2011<br>20-26/07/2014
Supportive depression	02/02/2011<br>10/10/2012	22-29/01/11<br>7-14/10/2012
Supportive schizophrenia	03/11/2011<br>27/05/2014	30/10-05/11/2011<br>25-31/05/2014

Supplementary Table 5. Twitter peaks and corresponding news search peaks in Google Trends between 01/01/2010 and 01/01/2015.

Search group	Keywords
Depression	depress[a-z0-9]*
Schizophrenia	schizophren[a-z0-9]*,<br>schizoaffective,<br>schizo affective,<br>schizo_affective,<br>schizo-affective,<br>#schizo[a-z0-9]*
Stigma	#mentalillness,<br>#endstigma,<br>#stigma,<br>#whatstigma,<br>#mhstigma,<br>#stigmahurts,<br>#mentalhealth[a-z0-9]*

Supplementary Table 6. Keywords used to search the Twitter API for depression, schizophrenia and mental health stigma-related posts.
